# The Anæsthetic Action of Electricity

**Published:** 1881-05

**Authors:** 


### THE ANÆSTHETIC ACTION OF ELECTRICITY

Is the subject of a lecture by Dr. Paul Julius Mœbius in the *Berliner klinische Wochenschrift.* He claims that the electric current rarely fails to alleviate not only neuralgic but even those pseudo-neuralgic pains caused by the compression of nerve roots from enlargement of adjacent inflamed organs. As instances of this relief he mentions caries of the spine, rheumatic spinal meningitis and phthisis. Similar results could be cited in painful affections of the muscles and joints. On this latter point he quotes the observations made by Brenner upon his own person, while suffering from acute articular rheumatism. In his case the shooting neuralgic pains were alleviated by the application of the electric current, while the inflammatory pains due to the inflammation itself remained. As further objects of the anæsthetizing effects of the current may be mentioned the toothache caused by caries, as well as the pains radiating from diseased uteri. Electricity acts, not like chloroform, nor like morphia; it is only effective in a limited number of cases, having a neuralgic character, a condition difficult to define. When however we encounter a characteristic neuralgic pain, its cause is to be sought for in a peculiar alteration of the sensitive nerves or central organs; this change we call neuralgic, which is not identical with the inflammatory, but cannot be more closely defined. This neuralgic change, which may occur with or without accompying inflammation is removed or altered by electricity. The galvanic current therefore serves the double purpose of a diagnostic and a therapeutic means; it alleviates pain while it proves that pain to be of a neuralgic nature. We have as yet no satisfactory theory based upon this experience to explain its purely anæsthetic action. As to the mode of application of the current, he found that any one of the usual modes, lessened the pain. The constant current acts anæsthetically more frequently than does the faradic; the anode is to be preferred to the cathode over the painful spots; slow interruption acts more effectively than the uninterrupted stream.

—*Med. Neuigk.*, No. 47, 1880.

